# The Integration of Multiple Nuclear-Encoded Transgenes in the Green Alga *Chlamydomonas reinhardtii* Results in Higher Transcription Levels

**DOI:** 10.3389/fpls.2019.01784

**Published:** 2020-02-14

**Authors:** Noam Shahar, Shira Landman, Iddo Weiner, Tamar Elman, Eyal Dafni, Yael Feldman, Tamir Tuller, Iftach Yacoby

**Affiliations:** ^1^ The George S. Wise Faculty of Life Sciences, School of Plant Sciences and Food Security, Tel Aviv University, Tel Aviv, Israel; ^2^ Department of Biomedical Engineering, The Iby and Aladar Fleischman Faculty of Engineering, Tel Aviv University, Tel Aviv, Israel; ^3^ The Sagol School of Neuroscience, Tel Aviv University, Tel Aviv, Israel

**Keywords:** copy-number variation, *Chlamydomonas reinhardtii*, Microalgae, the position-effect, droplet-digital PCR

## Abstract

The integration of genes into the nuclear genome of *Chlamydomonas reinhardtii* is mediated by Non-Homologous-End-Joining, thus resulting in unpredicted insertion locations. This phenomenon defines ‘the position-effect’, which is used to explain the variation of expression levels between different clones transformed with the same DNA fragment. Likewise, nuclear transgenes often undergo epigenetic silencing that reduces their expression; hence, nuclear transformations require high-throughput screening methods to isolate clones that express the foreign gene at a desirable level. Here, we show that the number of integration sites of heterologous genes results in higher mRNA levels. By transforming both a synthetic ferredoxin-hydrogenase fusion enzyme and a Gaussia-Luciferase reporter protein, we were able to obtain 33 positive clones that exhibit a wide range of synthetic expression. We then performed a droplet-digital polymerase-chain-reaction for these lines to measure their transgene DNA copy-number and mRNA levels. Surprisingly, most clones contain two integration sites of the synthetic gene (45.5%), whilst 33.3% contain one, 18.1% include three and 3.1% encompass four. Remarkably, we observed a positive correlation between the raw DNA copy-number values to the mRNA levels, suggesting a general effect of which transcription of transgenes is partially modulated by their number of copies in the genome. However, our data indicate that only clones harboring at least three copies of the target amplicon show a significant increment in mRNA levels of the reporter transgene. Lastly, we measured protein activity for each of the reporter genes to elucidate the effect of copy-number variation on heterologous expression.

## Introduction

Two main approaches are routinely used for transforming the green alga *Chlamydomonas reinhardtii*; the first includes integration into the chloroplast genome whilst the second includes integration into the nucleus. As opposed to its plastomic counterpart, nuclear transformation of *C. reinhardtii* is practically easier to perform and does not require selective cycles to obtain transgene homoplasmicity (Yamano et al., 2013). However, the integration of synthetic genes into the nuclear genome predominantly occurs randomly *via* Non-Homologues-End-Joining (NHEJ), leading to a large population of transformed cells with varied expression levels (Yamano et al., 2013; Weiner et al., 2018). Such phenomenon requires high-throughput screening methods for isolation of clones expressing the recombinant protein of interest at a desirable level, a non-trivial task that varies between target transgenes.

Such heterogeneity of nucleus-transformed clones is mainly explained by the “position effect”, a common phenomenon in which the expression of foreign genes is modulated by the genomic properties of their integration sites (León and Fernández, 2007; Zhang et al., 2014). It was previously shown that in order to average out position effects, one should collect at least 240 transformed colonies of *C. reinhardtii* (Lodha et al., 2008). Moreover, it was shown that the integration of plasmids occasionally results in deletions of genomic sequences, genomic rearrangements, and insertion of short DNA sequences (derived from fragmented DNA of lysed cells that are co-transformed along with the cassette) (Zhang et al., 2014; Jinkerson and Jonikas, 2015). Furthermore, gene silencing is an additional factor that plays a role in hindering heterologous expression in microalgae; it was shown to occur both at the transcriptional and post-transcriptional levels (Wu-scharf and Cerutti, 2000; Shaver et al., 2010), and is most likely part of an “immune system” protecting the cell against viral infections and transposable elements. Overall, the process of obtaining nuclear-transformed clones with a reasonably high expression of synthetic genes in *C. reinhardtii* is challenging.

In attempts to overcome these limitations, recent studies have developed different genetic approaches; for example, by performing an Ultraviolet (UV) mutagenesis on transformed clones with low transgene expression levels, two clones that further displayed a significant increase in the accumulation of the foreign gene were isolated (UVM-4 and UVM-11) (Neupert et al., 2009). Additional transformations into these clones resulted in a relatively high and uniform expression of heterologous genes (Barahimipour et al., 2015; Barahimipour et al., 2016). It was demonstrated that this unique phenotype of UVM-4 and UVM-11 is due to a gain-of-function mutation in the chromatin modification gene H4Ac, which most likely plays a key-role in repressing the chromatin state at the time of exogenous DNA integration (Barahimipour et al., 2016). Another approach used is the development of a detection system for measuring the frequency of homologues recombination (HR) events to successfully select clones with a desired integration site (Nour-Eldin et al., 2018). In yet another path, sequence optimization techniques were applied on target genes to mimic endogenous features of ORFs (e.g., the particularly high GC-content of the *Chlamydomonas* nuclear genome and the introduction of native introns into transgenes), and by that reducing the number of false clones (i.e., resistant to the selective pressure, but do not express the target transgene) and increase overall heterologous expression (Baier et al., 2018; Weiner et al., 2018). Furthermore, the development of the CRISPR/Cas9 genetic system in *C. reinhardtii* could potentially reduce the position-effect by “directing” the inserted transgene into a specific locus (Shin et al., 2016; Greiner et al., 2017).

Another important genetic feature that is known to create population diversity is the Copy-Number Variation (CNV); it is defined as a DNA segment found at variable copy quantities in a homogenic population of cells. CNV is linked to dozens of diseases (Redon et al., 2006; Malhotra and Sebat, 2012), but also serves as an important means to create and conserve population heterogenicity (Redon et al., 2006). In the context of synthetic biology, and especially in plants — where transgenes are mostly integrated randomly into the nuclear genome — it was shown that heterologous genes can be inserted more than once into different genomic locations, or as tandem repeats at a single locus to play either a positive or negative role on their expression (Stockhaus et al., 1987; Hobbs et al., 1990; De Block, 1993; Hobbs et al., 1993; Jorgensen et al., 1996; Que et al., 1997; Ku et al., 1999; Schubert et al., 2004; Wang et al., 2015; Zhang et al., 2015); while integration into distant loci mainly results in a synergistic effect (i.e., the expression is increased proportionally to its cognate DNA copy-number), repeat arrangements often induce transgene-silencing. However, a comprehensive study by Schubert et al., which was carried on 132 transgenic lines of *Arabidopsis thaliana* using three different reporters, emphasized the positive effect of copy-number on expression levels regardless to the genomic position and/or arrangement (repeats or inverted repeats) (Schubert et al., 2004). Generally, copy-number analysis in transgenic plants is carried out to select for lines that harbor a single copy of the integrated gene, as these typically segregate in a Mendelian fashion and are less prone to exhibit gene-silencing (Wang et al., 2015). Hence, such synthetic-CNV (sCNV) can be a major element that increases variation of nuclear transgenic lines in *C. reinhardtii* as well. For example, it was previously suggested that strategies that intentionally tend to increase transgenes copy-number may improve synthetic expression in *C. reinhardtii* (Lauersen et al., 2016). Another study indirectly approved the positive effect of transgenes copy-number by transforming *C. reinhardtii* with several different plasmids that contained the same synthetic gene, fused onto different fluorescence proteins (Wichmann et al., 2018). However, a large-scale analysis of sCNV has not yet been studied in *C. reinhardtii* or in any other microalga.

Today, several methods are used for copy-number analysis: (i) the traditional southern-blot, (ii) Quantitative PCR (qPCR), and (iii) droplet-digital PCR (ddPCR) (Southern, 1975; Schloss, 1990; Hindson et al., 2011; Wang et al., 2015; Zhang et al., 2015; Stefano et al., 2016; Collier et al., 2017). While southern-blot is a powerful tool (e.g., it provides additional information regarding the genomic organization and the integrity of the integrated cassette), it is arduous to use for a large number of samples, as it involves many laborious and time consuming steps, and was shown to yield unpredictable results when high number of DNA copies are examined (Collier et al., 2017). The recently developed ddPCR method was shown to detect the copy-number of genes as accurately as southern-blot analysis (Hindson et al., 2011; Pinheiro et al., 2012; Collier et al., 2017), and can be performed on a multiple number of samples simultaneously. Moreover, ddPCR has been optimized for CNV analysis in crop-plants (Demeke et al., 2014; Collier et al., 2017); for these reasons, we used ddPCR analysis to examine sCNV in *C. reinhardtii*.

In this work we studied the correlation between the number of DNA integration events, mRNA levels and expression levels in engineered strains of *C. reinhardtii*. For that purpose, we transformed both a synthetic ferredoxin-hydrogenase fusion enzyme (fd-hyd) (Eilenberg et al., 2016; Weiner et al., 2018) and a Gaussia-Luciferase reporter protein (gLuc) (Shao and Bock, 2008) into the nuclear genome of *C. reinhardtii*. We obtained 33 clones that exhibit a wide range of synthetic expression levels, and subsequently performed for each ddPCR to measure transgene DNA copy-number and mRNA levels. Finally, we measured protein activity for each of these clones to elucidate the impact of sCNV on heterologous expression.

## Materials and Methods

### Algal Cultures, Growth Conditions and Chlorophyll Determination

The algal cultures of the wild-type clone CC-124 and the hyda_1,2_ double-mutant (Meuser et al., 2012) were grown in Tris-Acetate-Phosphate (TAP) medium at 25°C under continuous cool daylight and cool white fluorescent lights (90 µmol m-2 s-1) stirring in 100 ml Erlenmeyers capped with silicone sponge enclosures. Experiments were performed when the cultures reached the density of 3*x*10^6^ cells/ml (as determined by Celeromics cell counter) that corresponds to chlorophyll concentrations of ~9 µg Chl/ml.

### *C. reinhardtii* Nuclear Transformation

DNA sequences were synthesized and cloned (using a recombination-based technique - CloneEZ™), into the pSL18::aph7 (Fischer and Rochaix, 2001) or pChlamy_1 (GeneArt™) plasmids, under the control of the endogenous *PSAD* or *HSP70A/RBCS2* promoters for fd-hyd and gLuc, respectively. 2 μg of linearized fd-hyd and gLuc plasmids were transformed by electroporation into 1.5*10^7^ cells of hyda_1,2_ double-mutant knockout strain and the wild-type strain CC-124, respectively, following the GeneArt *Chlamydomonas* Engineering Kit protocol (Life Technologies). Initial screening was carried out on Hygromycin (10 μg/ml).

### *Rhodobacter* *capsulatus* High Throughput Screen

Prepared precisely as described in (Wecker et al., 2011). Algal strains, overlaid with engineered H_2_-sensing *R. capsulatus*, were scanned using the Fuji FLA-5100 fluorescence imager. A 473 nm laser was used for excitation whereas 510 nm or 665 nm filters were used for quantifying GFP fluorescence and chlorophyll density, respectively.

### Luciferase *In Vivo* Assay

The protocol for Luciferase *in vivo* screening was adopted from (Mayfield and Schultz, 2004; Shao et al., 2007; Shao and Bock, 2008; Rasala et al., 2011) with the following modifications: the algal colonies were incubated for 10 minutes in dark, and then 10 µl of 0.01 mM Coelenterazine were dropped onto each colony. Finally, the plate was scanned for bioluminescence using the MicroChemi 6.0 camera.

### Nucleic Acids Extraction

One hundred milligrams of the cell pellet were taken for total RNA extraction using a RNeasy Plant Mini Kit (Qiagen 74903, http://www.qiagen.com/). One microgram of purified RNA from each sample was used for synthesis of complementary DNA (cDNA) using Applied Bio-System High-Capacity cDNA Reverse-Transcription Kit (with random primers). Thirty milligrams of the cell pellet were taken for total DNA extraction using an OMEGA E.Z.N.A SP Plant DNA kit. DNA and RNA concentrations were quantified by a Thermo Scientific™ NanoDrop™ 2000 Spectrophotometer.

### Droplet Digital PCR

The ddPCR reaction mixture was prepared from a QX200™ ddPCR™ EvaGreen Supermix, 250 nM primers, 5 ng of cDNA/1 ng of DNA template (the cDNA quantity was based on the initial amount of RNA concentration used for the reverse-complementation) and DDW to a total volume of 22 μl. Each reaction mixture was loaded into a DG8™ Cartridge (Bio-Rad), and a volume of 70 μl of QX200™ Droplet Generation Oil was loaded into the oil-well of each channel. The DG8™ Cartridge was then placed into the QX200™ Droplet Generator. Next, 40 μl of the created droplets were transferred to a ddPCR™ 96-Well Plate. The plate was heat-sealed with a foil seal and further undergone PCR using C1000 Touch™ Thermal Cycler. Subsequently, the 96-well PCR plate was placed onto the QX200™ Droplet Reader. Further analysis of the data was performed using QuantaSoft™, where the absolute concentration of each target (fd-hyd or gLuc) was normalized to the absolute quantification of the reference gene *CBLP*. The primers used were:

- fd-hyd (5'- TCGCCATGCTGGAGAAGTC -3', 5'- AGCTGGACACGTAGGGGAT -3');
- gLuc (5'- CCCAAGTGGACCTGTGTGTG -3', 5'- CACTTCTTCAGCAGGTCGCTA -3');
- *CBLP* (5'- AAAGCCACGGACAGCACATC-3', 5'- GATGGCCAGTTCTGCCTGAC-3').

### fd-hyd Induction

Anaerobic induction was carried out as follows: liquid cell cultures (40 ml) at mid log phase (3*x*10^6^ cells/ml) were concentrated by centrifugation (2,800 g for 7 min) and re-suspended in 4 ml of TAP HEPES buffer (50 mM HEPES, pH 7.8). The concentrated cells were transferred into septum stopper sealed 14 ml glass serum vials (Wheaton), covered by aluminum foil and purged for 30 min with Argon at room temperature. Thereafter, the concentrated cultures were incubated in dark, at room temperature, under 60 rpm agitation for additional 90 minutes.

### Hydrogenase Activity Assay by Methyl-Viologen

Carried out precisely as described in (Eilenberg et al., 2016). Following two hours of dark anaerobiosis, cells were transferred into a buffer containing reduced methyl viologen and Triton-X for lysing the cells. A 500 µl sample was drawn from the headspace and the H_2_ concentration was determined by gas chromatography. The amount of enzyme was calculated based on the constant fd-hyd specific activity (Yacoby et al., 2011).

### Statistical Analysis

#### Pearson Correlation

Pearson correlation coefficients (rho) and p-values were calculated using python's “scipy.stats.pearsonr” function.

#### Two-Way ANOVA

To determine whether the effect of both the reporter type (fd-hyd or gLuc) and the DNA copy-number on the mRNA levels is significant, a two-way ANOVA test was carried out using SPSS.

#### Tukey’s Range Test

To determine which of the copy-number group mRNA levels means are statistically different from each other, a Tukey's range test was performed using SPSS.

## Results

All data are summarized in **Table S1**.

### Construction and Isolation of an Array of Clones With Varied Expression Levels

**Figure 1:**
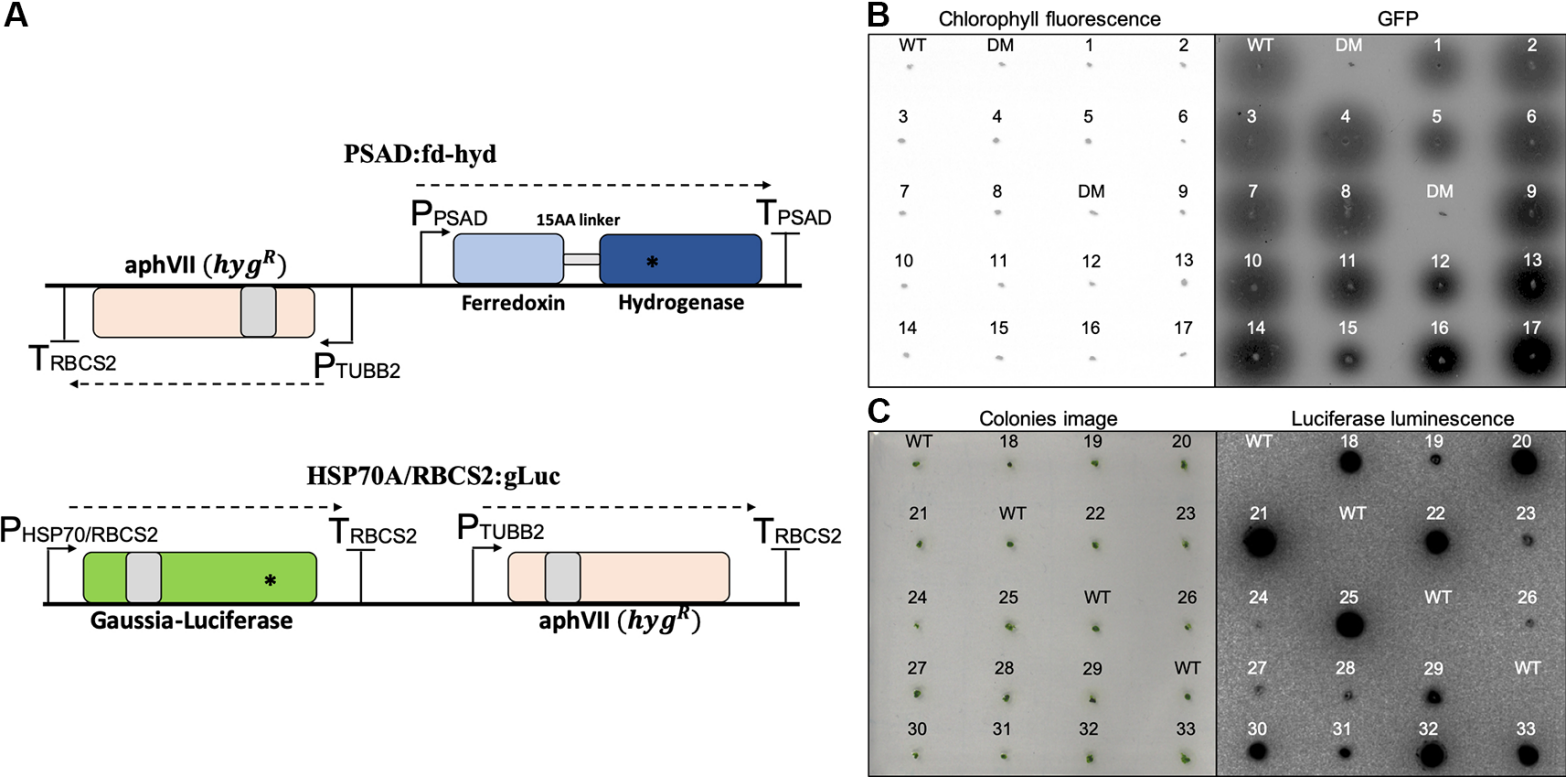
Construction and selection of positive transformants expressing the reporter genes. **(A)** Plasmid maps of the fd-hyd (upper) and the gLuc (bottom) reporter genes. The fd-hyd transgene was cloned into a modified pSL18 vector (the *paro^R^* was replaced by the *hyg^R^*) under the control of the PSAD regulatory sequences. The gLuc transgene was cloned into pChlamy_1 plasmid (*hyg^R^*) under the control of the HSP70A/RBCS2 promoters and the RBCS2 terminator. Gray boxes represent the first intron of the RBCS2 within the coding-sequences. Asterisks (*) represent the ddPCR amplicon loci of fd-hyd (51bp) and gLuc (85 bp). The cut site of *ScaI* is found outside the amplicon area (not shown). **(B)** *Rhodobacter* assay for selected hydrogen producing colonies. fd-hyd colonies were overlaid with engineered H_2_-sensing *R. capsulatus* and were scanned using a 473 nm laser for excitation whereas 510 nm or 665 nm filters were used for quantifying GFP fluorescence (right) and chlorophyll density (left), respectively. **(C)** *in-vivo* Luciferase assay for the gLuc selected colonies; gLuc colonies were incubated for 10 min in dark, and then 10 μl of 0.01 mM Coelenterazine were dropped onto each colony. The left panels refer to either the detection of the colonies chlorophyll **(A)** or the photographs of colonies prior to luminescence imaging **(B)**. The right panels refer to GFP fluorescence emitted by *R. capsulatus* (A) or luminescence emitted by gLuc **(B)**. WT = CC-124 wild-type strain (which naturally produces hydrogen), DM = hyda_1,2_ double-mutant strain (does not produce hydrogen). The numbers refer to clone indices.

To obtain clones with a wide-range of synthetic expression levels, we took advantage of the random integration predominately occurring in the nuclear genome and performed two transformations, distinguished by the promoter, 5'UTR, reporter gene, 3'UTR and the presence of an intron (see supplemented ‘DataSheet 1_v1’ for GenBank files of full annotated plasmid sequences); (i) the _p_ PSAD:fd-hyd cassette contains an optimized Hydrogenase (HYDA1) enzyme fused to an N-terminal Ferredoxin gene under the control of the native Photosystem I reaction center subunit II (PSAD) regulatory sequences. It was transformed into a mutant strain of *C. reinhardtii* which shows a negligible production of gaseous hydrogen (*the hyda*_1_ and *hyda*_2_ double-mutant strain) in order to screen for colonies that restored their ability to produce hydrogen. (ii) the _p_ HSP70A/RBCS2:gLuc plasmid, which is comprised of an optimized Gaussia-Luicferase gene under the control of the native Heat-Shock Protein 70A promoter fused to the promoter of the Rubisco Small subunit 2 (RBCS2), and contains the first intron of the RBCS2 gene. It was transformed into the wild-type CC-124 strain (**Figure 1A**, *Algal Cultures, Growth Conditions and Chlorophyll Determination* and *C. reinhardtii Nuclear Transformation*, the ratio of plasmid to cells used is $\frac{1\;\mathrm{ng}}{10,000\;\mathrm{cells}}$). For each construct, we obtained 100 colonies for further analysis. To screen for hydrogen-producing colonies, we used an engineered *Rhodobacter capsulatus* that expresses GFP in the presence of gaseous hydrogen (Wecker et al., 2011; Wecker and Ghirardi, 2014) (**Figure 1B**, *Rhodobacter capsulatus High Throughput Screen*). To screen for gLuc positive colonies, we used a Luciferase *in vivo* assay, as described in (Shao and Bock, 2008) (**Figure 1C**, *Luciferase In Vivo Assay*). A total of 33 positive clones (17 fd-hyd and 16 gLuc, ~16% out of the initial collected pool) were collected for further analysis.

### DNA Copy-Number and mRNA Level Quantifications

**Figure 2:**
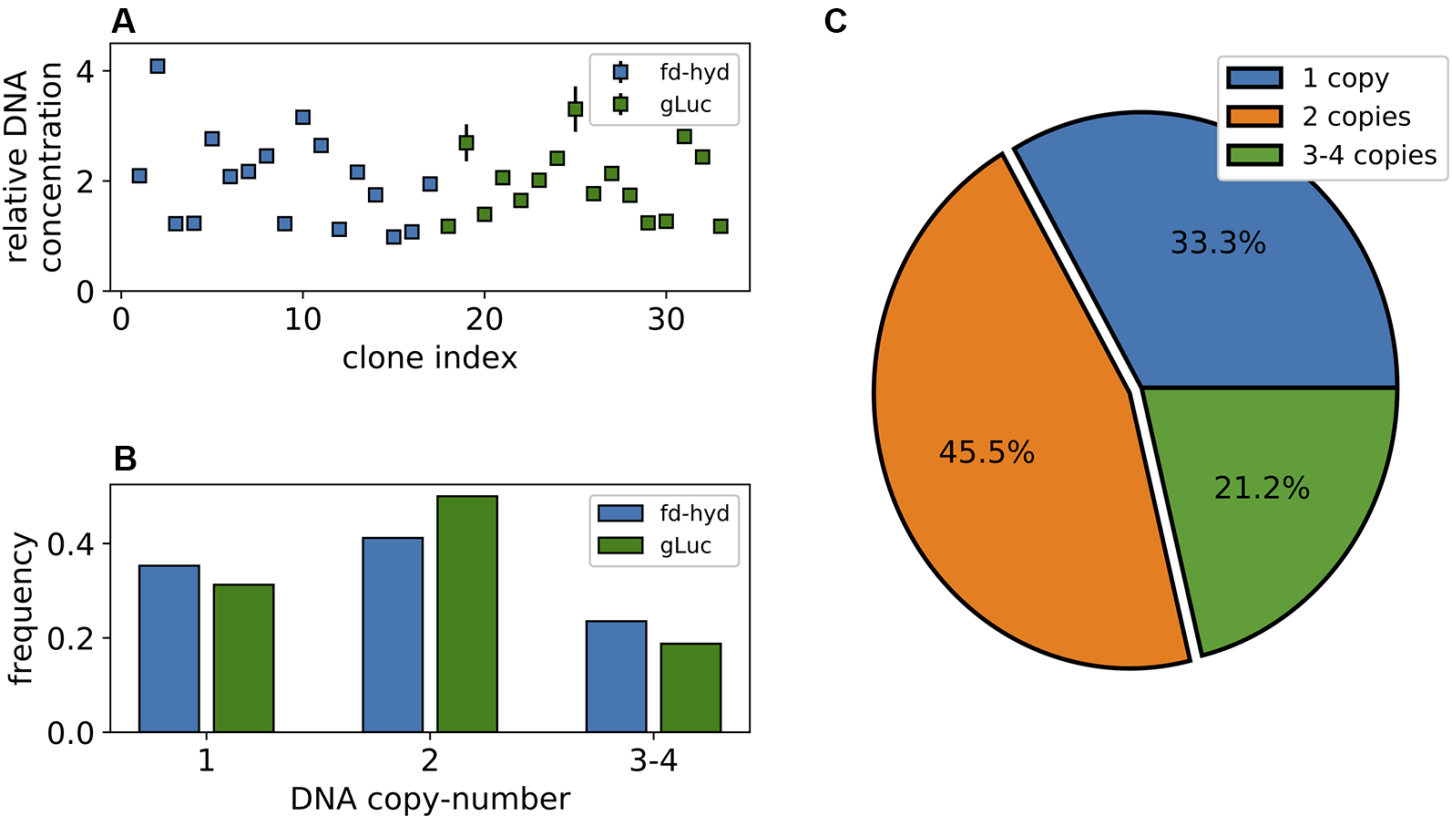
Synthetic copy-number variation of the fd-hyd and the gLuc transgenic clones. **(A)** Transgenes DNA copy-number in 33 nucleus-transformed clones. Copy-number values are calculated as the ratio of the target's concentration to the reference gene (*CBLP*) concentration. The error bars represent the maximum and minimum values of Poisson distribution for the 95% confidence interval, as given by the QuantaSoft™ software. **(B)** The ratio of clones found in each copy-number group separated by the reporter gene. **(C)** Distribution of transgenes' copy-number in all clones.

To quantify the number of transgenes integration events that occurred in each of the fd-hyd and the gLuc expressing clones, we extracted total DNA and performed digestion using the restriction enzyme *ScaI*, in order to exclude the possibility that tandem target duplications or targets found in the same chromosome would be encapsulated by the same droplet (*Nucleic Acids Extraction,* the cut site was located outside the amplicons). Next, we used the digested DNA template of each clone for two independent ddPCR reactions using the EvaGreen Supermix; the first reaction amplified an amplicon within the target reporter gene (fd-hyd or gLuc) and the second reaction amplified an amplicon within a reference gene, known to harbor a single copy in the *C. reinhardtii* genome (*CBLP)* (Schloss, 1990). The number of target molecules per µl (‘concentration’, retrieved by QuantaSoft™), obtained for each target gene was divided by the concentration obtained for the reference gene (**Figure 2A**); this ratio provided a non-integer number, which was further rounded to obtain the target's DNA copy-number (*Droplet Digital PCR*). Importantly, we did not detect major differences in the sCNV distribution between the fd-hyd and the gLuc expressing clones (**Figure 2B**), thus excluding the possibility that the sequence composition of the coding-sequence or the regulatory elements affects the integration efficiency in our conditions. Interestingly, we observed that the most frequent number of integration events is two (45.5%), whereas one integration site was found in 33.3% of all clones (**Figure 2C**). Additionally, 18.1% and 3.1% of clones contained three and four target copies, respectively (**Figure 2C**).

**Figure 3:**
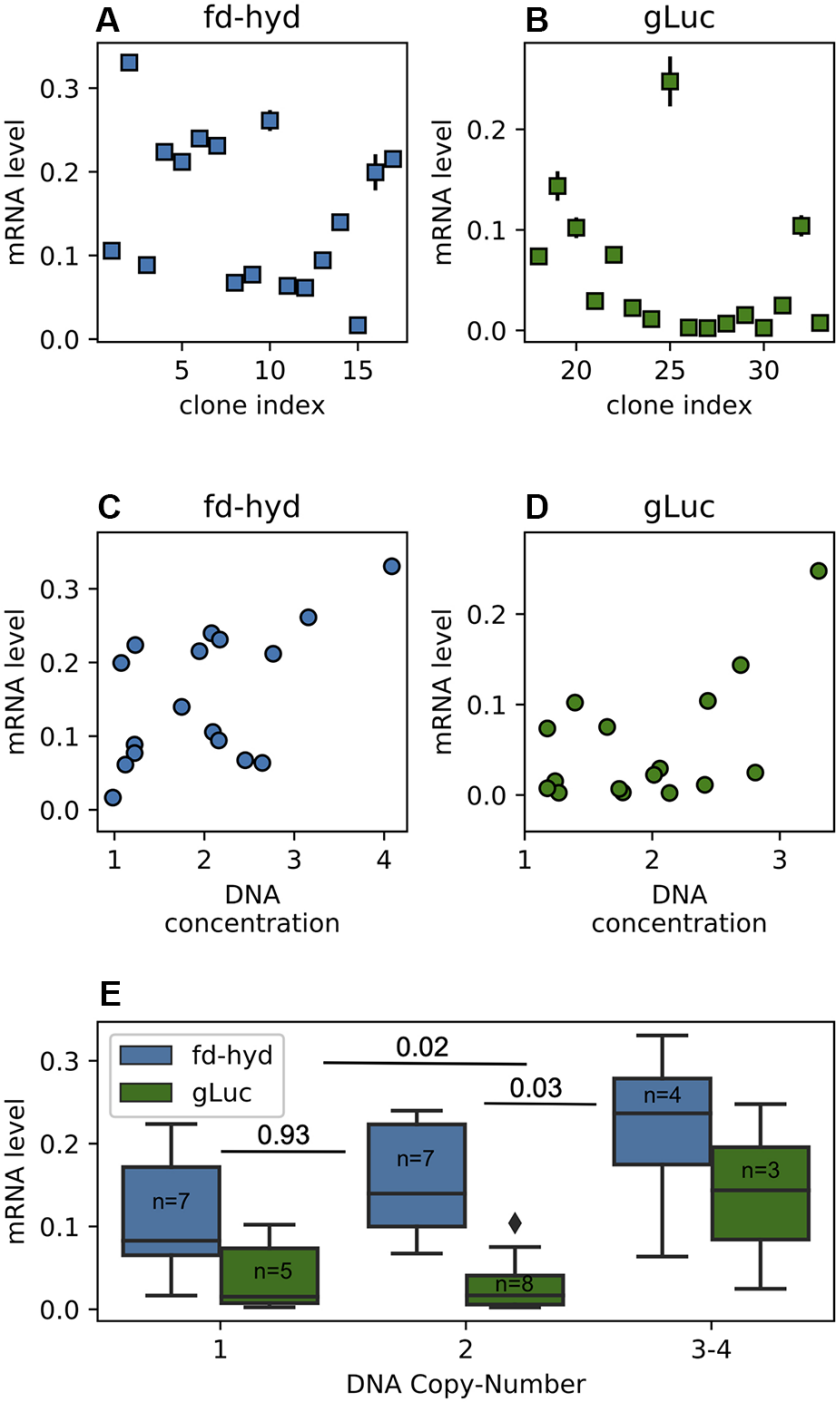
Transgenes mRNA abundance in the fd-hyd and the gLuc clones. **(A**, **B)** Transgenes relative mRNA abundance in the fd-hyd and the gLuc clones, respectively. The mRNA levels are calculated as the ratio of the target's concentration to the reference gene (*CBLP*) concentration. The error bars represent the maximum and minimum values of Poisson distribution for the 95% confidence interval, as given by the QuantaSoft™ software. **(C**, **D)** The effect of the relative raw DNA concentration (non-integer values) on transcription levels in the fd-hyd and the gLuc clones, respectively. Pearson correlation coeffcients are 0.56, p = 0.02 and 0.55, p = 0.027 for the fd-hyd and the gLuc clones, respectively. **(E)** The effect of the DNA copy-number on transcription levels. P-values were calculated using two-way ANOVA post-hoc test, and are shown between each copy-number group. The line within each box represents the median. The number of samples for each group is indicated within each box. The copy-number group “3-4” also contains one clone that harbors a copy-number of four (fd-hyd clone #2).

To quantify the mRNA levels for the fd-hyd clones we anaerobically incubated the cultures (in order to induce the fd-hyd expression for the later Hydrogenase activity assay, which was sampled from the same cultures), extracted total RNA and used a similar ddPCR analysis (without template restriction), normalized by the same reference house-keeping gene *CBLP* (Schloss, 1990) (*Nucleic Acids Extraction, Droplet Digital PCR, fd-hyd Induction*, **Figure 3A**). The same procedure was carried out on the gLuc clones, without anaerobic incubation (**Figure 3B**). Although our results show that the gLuc transcript levels are generally lower than the fd-hyd mRNA abundance, we observed a significant correlation between the raw DNA copy-number values (the non-integer numbers received by the ratio of target-to-reference concentration) to the mRNA levels in each reporter separately (**Figures 3C, D** and *Pearson Correlation*; Pearson's rhos are 0.56, p = 0.02 and 0.55, p = 0.027 for the fd-hyd and the gLuc clones, respectively), indicating the positive effect of a transgene copy-number on its transcription level. Moreover, by grouping the mRNA levels to their cognate copy-number values (and by that reducing the variation created by the position effect), we observed a significant difference in transcription between the copy-number groups both in the fd-hyd and the gLuc clones (*Two-Way ANOVA*; 2-way ANOVA p = 0.002). Subsequently, we detected a significant separation between two-copies to ≥ three-copies groups, whereas the difference between one-copy to two-copies did not yield a significant difference (**Figure 3E** and *Tukey's Range Test*; Tukey's range test p = 0.03 and p = 0.93, respectively).

### Quantification of Protein Activity

**Figure 4:**
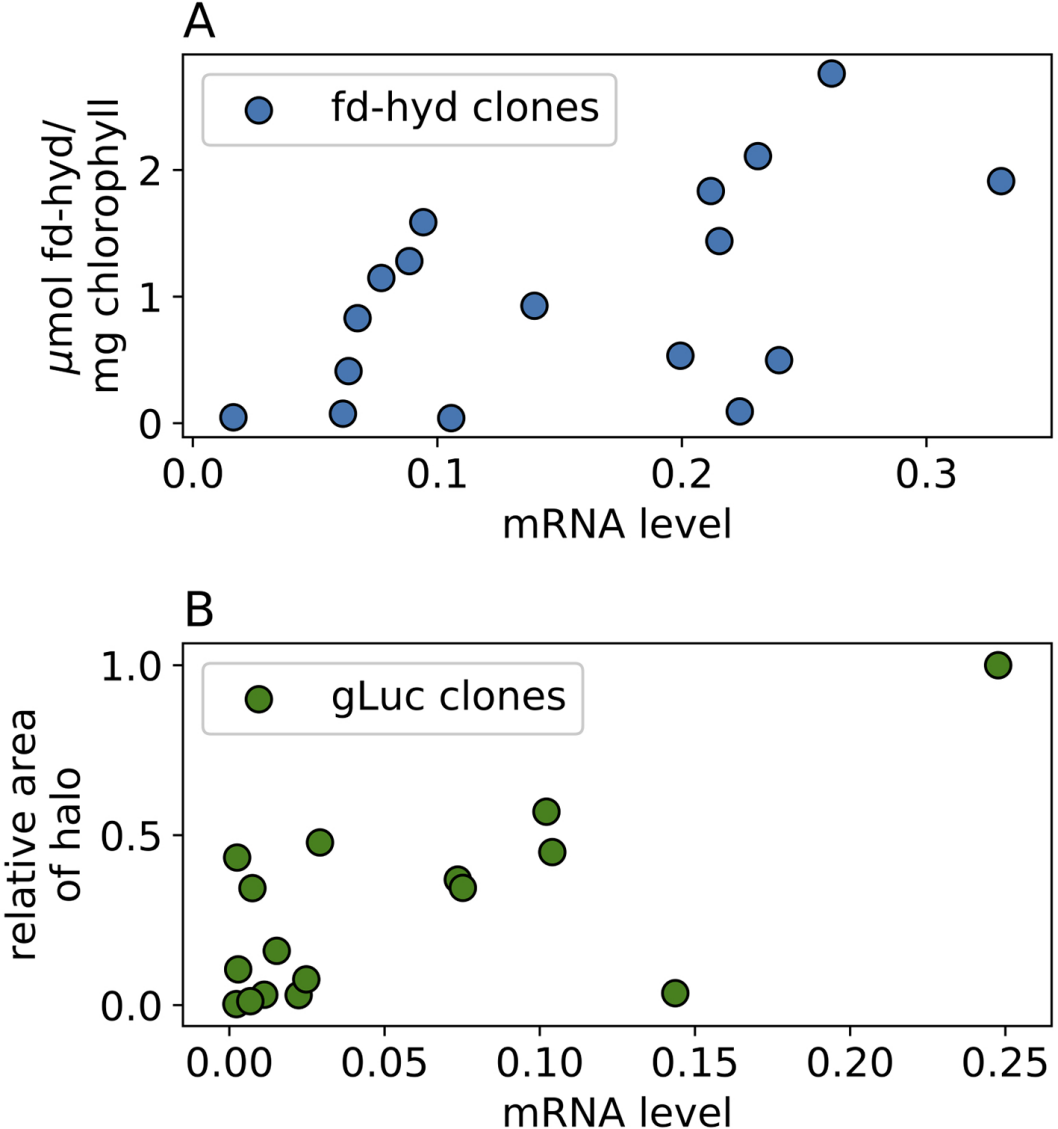
The effect of mRNA levels on protein abundance in the fd-hyd and the gLuc clones. **(A)** the fd-hyd protein activity was measured using MV. The values are $\frac{\mu \text{mol fd}-\text{hyd}}{\text{mg chlorophyll}}\text{ X }10^{6}$ **(B)** gLuc protein values were calculated using CFQuant's “Area” value normalized to the colony size (Dafni et al., 2019). Pearson rhos are 0.55, p = 0.023 and 0.69, p = 0.003 for the fd-hyd and the gLuc clones, respectively. All values show mean of three biological repeats.

To measure the fd-hyd active protein abundance we used the MV quantiﬁcation assay (*Hydrogenase Activity Assay by Methyl-Viologen*) on three biological repeats (**Table S1**). To measure the gLuc reporter activity, we plated the colonies onto three independent plates while shuffling positions (**Figure S1**, **Table S1**) and performed a Luciferase *in-vivo* assay (Mayfield and Schultz, 2004; Rasala et al., 2011). To calculate the relative protein activity we used the image analysis software CFQuant (https://www.energylabtau.com/cfquant) (Dafni et al., 2019), and obtained the values of “Area” (i.e., sum of pixels) divided by the colony size. In order to reduce the variation between plates even further, these values were divided to the highest value within the same plate; this also allowed us to retrieve a normalized quantity which can further be averaged and compared between plates. We observed a strong positive correlation between the mRNA and protein activity in both constructs (**Figures 4A, B**, Pearson rhos are 0.55, p = 0.023 and 0.69, p = 0.003 for the fd-hyd and the gLuc clones, respectively). Finally, the direct correlation of the raw DNA values to protein activity levels is significant in the fd-hyd clones, whereas in the gLuc clones this effect is weak (**Figure S2**, Pearson rhos are 0.61, p = 0.009, and 0.11, p = 0.67 for the fd-hyd and the gLuc clones, respectively).

## Discussion

In this work, we suggest that in addition to already known factors (e.g., position-effects, epigenetic-silencing, location in 3D genomic organization, chromatin state, local DNA folding, interruption of native genes), the number of integration sites for a synthetic gene transformed into the nucleus of *C. reinhardtii* is yet another important feature that affects its transcription level and may contribute to the expression-heterogeneity between transgenic clones. By accurately measuring the number of integration sites of two different reporter genes in 33 nucleus-transformed clones, we revealed that the transformed transgene is usually integrated within one to two loci (~79%), whereas in most cases it is inserted two times (**Figure 2C**, based on the plasmid-to-cells ratio of 1:10,000). We observed that the two constructs used in this study show roughly the same distribution of DNA copy-number in the genome, even though they vary in the type of gene, sequence composition, presence of introns and the regulatory elements (**Figure 2B**). Importantly, because fragmented plasmids (that may carry the resistance gene solely) may potentially integrate the genome at the time of transformation, we chose to assess the copy-number based on amplicons that are found within the reporter transgene sequences themselves.

To understand the effect of transgene copy-number on transcript levels, we measured the mRNA abundance in the transgenic clones. We were able to show that transgene DNA copy-number positively correlates with its conjugated mRNA levels, with the major effect of three and four copies (**Figures 3C–E**). However, it is critical to note that some clones with multiple integration sites exhibit low transcription levels, and vice-versa (i.e., some clones with a single copy-number show relatively high mRNA levels, **Figures 3C, D**); these exceptions may be explained by other factors (in which their degree varies between transgenes) that can potentially affect transcription; for example (i) the “position-effect”, (ii) gene-silencing, (iii) integration of fragmented plasmids, and/or (iv) antagonistic effects of multiple insertion sites. Importantly, these “outliers” seem to be more frequent in the gLuc clones, a fact that together with their global reduction in mRNA levels (**Figures 3A, B**) emphasizes the notion that the nature of the sequence (e.g., regulatory elements, presence of introns, CDS properties) does affect transcription features. In order to reduce the effect (or “noise”) of the above-mentioned factors we presented the data as averages of level of expression, separated by the copy-number groups (**Figure 3E**). Based on our experimental conditions, three integration events had the highest impact on transcription. According to this, one can estimate that multiple DNA copies of the transgene do not synergistically affect transcription (as a major part of transgene copies would not be expressed at all); alternatively, it can be hypothesized that the higher the transgene's copy-number is, the chances of at least one cassette to integrate into a highly expressed DNA region are increased. With that, it is tempting to use sCNV analysis in order to screen for highly expressing clones in *C. reinhardtii* (when no efficient screening method exists) by searching for clones that harbor at least three copies of the integrated transgene.

Finally, we measured the recombinant protein abundance (as a function of protein activity) in our library (**Figures 4A, B**); the results align with earlier evidences, where the correlation between mRNA to protein levels in the nuclear genome of *C. reinhardtii* is high (Baier et al., 2018; Weiner et al., 2018). Yet, several lines show notable exceptions from this rule (e.g., clones with high mRNA levels show low/negligible levels of protein); these results may be explained by either post-transcriptional mechanisms of gene-silencing, and/or deletions or mutations at the synthetic gene regulatory elements (which may reduce translation efficiency), or at the CDS region (which may produce non-mature mRNAs, or yield a non-functional protein). Lastly, the direct effect of DNA copy-number on protein levels in the fd-hyd clones is significantly sound (**Figure S2A**); however, the same analysis yielded poor results on the gLuc clones (**Figure S2B**). As mentioned above, as the mRNA levels of the gLuc lines are less affected by their cognate DNA copy-number, let alone the protein levels - which are subject to more regulations (e.g., post-transcriptional and post-translational modifications).

## Data Availability Statement

All datasets generated for this study are included in the article/**Supplementary Material**.

## Author Contributions

NS and IY designed the research. NS, SL, IW, TE, and YF performed the experimental procedures. NS, IW, and ED performed the computational procedures. NS, IW, TT and IY wrote the paper.

## Funding

The single source of this research is Israel Science foundation grant number 1646/16.

## Conflict of Interest

The authors declare that the research was conducted in the absence of any commercial or financial relationships that could be construed as a potential conflict of interest.
