# Supplementary material for: The Integration of Multiple Nuclear-Encoded Transgenes in the Green Alga Chlamydomonas reinhardtii Results in Higher Transcription Levels
Source: Front Plant Sci. 2020 Feb 14;10:1784. doi: 10.3389/fpls.2019.01784 (PMC7033495; doi:10.3389/fpls.2019.01784)
Supplement: Supplementary file 2 [file DataSheet_2.docx]

# **Supplemental information**

## **Supplementary Figure S1. Quantification of the gLuc reporter activity.** gLuc clones were picked onto three independent plates while their positions shuffled. Then, a Luciferase *in-vivo* assay was performed on each plate. The calculation of the relative protein abundance was done using the image analysis software CFQuant (Dafni et al. 2019). The number beneath each colony represents the colony index and its DNA copy-number (index_CN).

## **Supplementary Figure S2. The effect of DNA copy-number on protein activity in the fd-hyd and the gLuc clones. (A)** the fd-hyd protein abundance was measured using MV. The values are $\frac{\mu mol fd-hyd}{mg chlorophyll} X {10}^{7}$. **(B)** gLuc protein values were calculated using CFQuant’s “Area” value (Dafni et al., 2019). Pearson rhos are 0.61, p=0.009 and 0.11, p=0.67 for the fd-hyd and the gLuc clones, respectively. All protein values show mean of three biological repeats.

**Supplementary Table S1.** For each clone number (“Clone ID” column) all values of DNA, RNA and protein levels are shown. The “DNA measurement” and “RNA measurement” represent the droplet-digital PCR results, as obtained from the QuantaSoft software. “Conc” columns indicate the concentration of the amplicon as $\frac{\mathrm{molecules}}{\mathrm{uL}}$, whereas the “confMax” and “confMin” signify the maximum and minimum values of Poisson distribution for the 95% confidence interval. Columns of “Target” and “Ref” represent the raw values obtained for the reporter genes (fd-hyd or gLuc) and a reference gene (*CBLP*), respectively, whereas the “Ratio” columns refer to the ratio of $\frac{\mathrm{Target}}{\mathrm{Ref}}$ . The “Copy-Number” column is the rounded numbers of “Ratio_Conc” and represents the final copy-number group. The “Protein measurement” columns show the values of three independent measurements either for the fd-hyd reporter (values are calculated by MV, $\frac{\mu mol fd-hyd}{mg chlorophyll}$) or the gLuc reporter (“Area” values obtained by CFQuant).

## Supplemental reference:

Dafni, E., Weiner, I., Shahar, N., Tuller, T. & Yacoby, I. 2019. Image-Processing Software for High-Throughput Quantification of Colony Luminescence. *mSphere*.
